# Supplementary material for: Splice-Junction-Based Mapping of Alternative Isoforms in the Human Proteome
Source: Cell Rep. Author manuscript; Available in PMC 2020 Jan 15. (PMC6961840; doi:10.1016/j.celrep.2019.11.026)
Supplement: 3 [file NIHMS1546469-supplement-3.zip › DF2/PXD0010154/Liver-304-Q02410-IKAPEGESQPMTEVDLFISTQR.pdf]

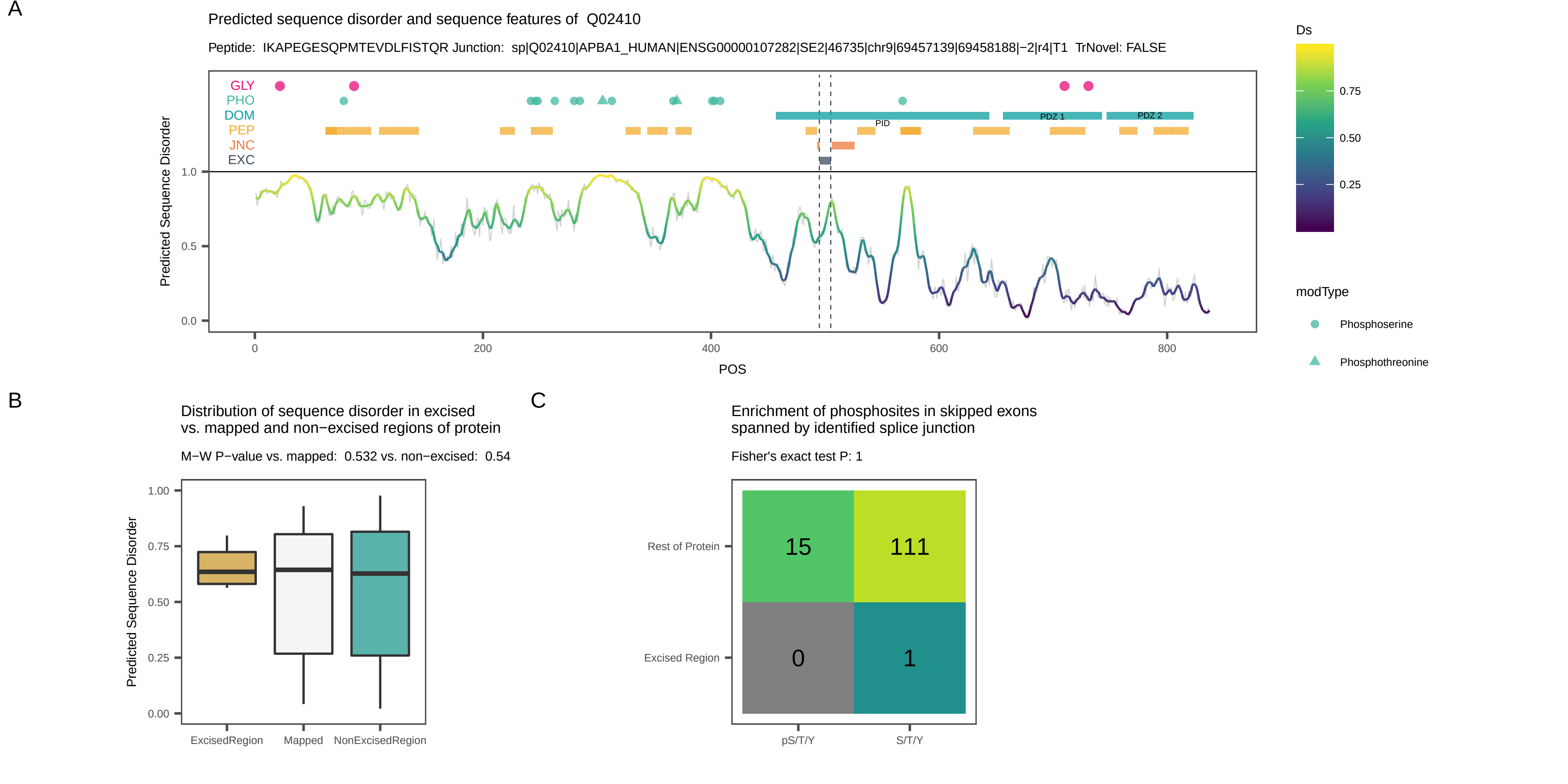
A
Predicted sequence disorder and sequence features of Q02410
Peptide: IKAPEGESQPMTEVDLFISTQR Junction: sp|Q02410|APBA1_HUMAN|ENSG00000107282|SE2|46735|chr9|69457139|69458188|−2|r4|T1 TrNovel: FALSE
GLY
PHO
DOM
PEP
JNC
EXC
PID
PDZ 1
PDZ 2
Predicted Sequence Disorder
1.0
0.5
0.0
0
200
400
600
800
POS
Ds
0.75
0.50
0.25
modType
Phosphoserine
Phosphothreonine
B
Distribution of sequence disorder in excised
vs. mapped and non−excised regions of protein
M−W P−value vs. mapped: 0.532 vs. non−excised: 0.54
Predicted Sequence Disorder
1.00
0.75
0.50
0.25
0.00
ExcisedRegion
Mapped
NonExcisedRegion
C
Enrichment of phosphosites in skipped exons
spanned by identified splice junction
Fisher's exact test P: 1
Rest of Protein
Excised Region
15
111
0
1
pS/T/Y
S/T/Y
